# Genomic profiling of idiopathic peri-hilar cholangiocarcinoma reveals new targets and mutational pathways

**DOI:** 10.1038/s41598-023-33096-0

**Published:** 2023-04-24

**Authors:** Leonard M. Quinn, Sam Haldenby, Philip Antzcak, Anna Fowler, Katie Bullock, John Kenny, Timothy Gilbert, Timothy Andrews, Rafael Diaz-Nieto, Stephen Fenwick, Robert Jones, Eithne Costello-Goldring, Graeme Poston, William Greenhalf, Daniel Palmer, Hassan Malik, Chris Goldring

**Affiliations:** 1 Department of Pharmacology and Therapeutics, Institute of Systems, Molecular and Integrative Biology, Sherrington Building, University of Liverpool, Liverpool, UK; 2 Centre for Genomic Research, University of Liverpool, Liverpool, UK; 3 Computational Biology Facility, University of Liverpool, Liverpool, UK; 4 Department of Health Data Science, University of Liverpool, Liverpool, UK; 5 Liverpool University Hospitals NHS Foundation Trust, Liverpool, UK

**Keywords:** Gastroenterology, Molecular medicine, Oncology

## Abstract

Peri-hilar cholangiocarcinoma (pCCA) is chemorefractory and limited genomic analyses have been undertaken in Western idiopathic disease. We undertook comprehensive genomic analyses of a U.K. idiopathic pCCA cohort to characterize its mutational profile and identify new targets. Whole exome and targeted DNA sequencing was performed on forty-two resected pCCA tumors and normal bile ducts, with Gene Set Enrichment Analysis (GSEA) using one-tailed testing to generate false discovery rates (FDR). 60% of patients harbored one cancer-associated mutation, with two mutations in 20%. High frequency somatic mutations in genes not typically associated with cholangiocarcinoma included *mTOR, ABL1* and *NOTCH1*. We identified non-synonymous mutation (p.Glu38del) in *MAP3K9* in ten tumors, associated with increased peri-vascular invasion (Fisher’s exact, p < 0.018). Mutation-enriched pathways were primarily immunological, including innate Dectin-2 (FDR 0.001) and adaptive T-cell receptor pathways including PD-1 (FDR 0.007), CD4 phosphorylation (FDR 0.009) and ZAP70 translocation (FDR 0.009), with overlapping HLA genes. We observed cancer-associated mutations in over half of our patients. Many of these mutations are not typically associated with cholangiocarcinoma yet may increase eligibility for contemporary targeted trials. We also identified a targetable *MAP3K9* mutation, in addition to oncogenic and immunological pathways hitherto not described in any cholangiocarcinoma subtype.

## Introduction

Cholangiocarcinoma constitutes a heterogeneous group of aggressive malignancies arising in the biliary tract epithelium and accounts for ~ 2% of all cancer-related deaths worldwide annually^1^. Surgery may be curative when technically feasible, but the disease is often advanced at presentation and chemo-refractory in nature, with 5-year overall survival remaining dismal (< 10%)^2^. Novel cytotoxic and immunotherapeutic strategies are desperately needed.

Western cholangiocarcinoma is idiopathic in origin and sharply contrasts Asia, where the disease is more common and primarily associated with liver fluke and viral hepatitis infections^3^. Anatomically divided into intra-hepatic (iCCA), peri-hilar (pCCA) and distal subtypes (dCCA), peri-hilar cholangiocarcinoma constitutes ~ 60% of cases^4^. All three subtypes share common somatic mutations, such as *TP53* and *KRAS*, but significant less frequent subtype specific mutations have been identified with implications for targeted therapies^5^. iCCA has been extensively sequenced leading to breakthrough clinical trials of efficacious targets unique to that subtype, including *IDH1* mutations^6^, and *FGFR2* fusions^7^.

Despite its predominance, pCCA has undergone little next generation sequencing comparative to both iCCA and dCCA. Targeted studies that have included western pCCA often consider it under the umbrella of extra-hepatic cholangiocarcinoma, combining peri-hilar data with dCCA. Previous exploratory DNA sequencing studies in pCCA such as whole exome, have been limited to Asian populations with infective etiologies. International studies combining eastern and western cohorts, are limited to targeted sequencing in the western participants^8,9^. The results of Asian whole exome studies are not applicable to Western idiopathic pCCA populations and potential disparity may limit eligibility for new and existing trials of targeted therapies. Furthermore, limited understanding of the mutational landscape in idiopathic pCCA may impact tumor immunology, critical given that responses to immune checkpoint inhibition in cholangiocarcinoma have been disappointing^10^.

To address these issues, we performed a comprehensive genomic analysis of 42 surgically resected U.K. pCCA tumors to shed light on the mutational biology of idiopathic western pCCA and identified multiple new targets and pathways which warrant further investigation.

## Methods

### Tissue collection

pCCA was defined anatomically as a tumor of the extrahepatic biliary tree, arising proximal to the cystic duct insertion. Twenty-five surgically resected formalin-fixed-paraffin-embedded pCCA tumors, dating August 2010 to August 2016 (together with paired histologically normal bile ducts), were obtained for whole exome sequencing. Seventeen fresh snap frozen surgically resected tumors were collected prospectively between August 2017 and August 2019 for targeted sequencing.

All samples underwent microdissection and Consultant Histopathologist review to ensure adequate tumor cellularity > 50% with staging according to the American Joint Committee on Cancer Classification (8th edition).

### DNA extraction, quality control, library preparation and whole exome sequencing

DNA was extracted using DNEasy (Qiagen, Venlo, Netherlands). FFPE-derived DNA samples were quantified by Qubit double stranded DNA high sensitivity assay (Thermofisher Scientific, Waltham, Massachusetts, USA). An Agilent Next Generation Sequencing FFPE QC kit (Agilent Technologies, Santa Clara, California, USA) was used to quantify and qualify the DNA by qPCR with exome sequencing on Illumina HiSeq 4000 (version 1 chemistry) (Illumina, San Diego, California, USA), generating 2 × 150 base pair paired end read libraries. Targeted hotspot AmpliSeq libraries were prepared using Ion AmpliSeq Library Kit 2.0 and Ion AmpliSeq Cancer Hotspot Panel v.2 (Thermofisher) with sequencing performed on Ion PGM Sequencing 200 kit V2.

### Initial bioinformatics processing and quality assessment

Basecalling and de-multiplexing of indexed reads was performed by CASAVA 1.8.2 (Illumina). Raw FASTQ files were trimmed to remove adapter sequences using Cutadapt version 1.2.1 (RRID:SCR_011841). Low quality reads were removed using Sickle version 1.200 (RRID:SCR_006800) with a minimum window quality score of 20. After trimming, reads shorter than 20 bp were removed.

### Variant calling and annotation

Reads were aligned to the human reference genome sequences (GRCh38) using Burrows Wheeler Alignment version 0.7.5a. Alignments were filtered to remove reads with a mapping quality < 10. Mapped reads were locally realigned using the Genome Analysis Tool Kit version 2.1.13. Read duplicates were identified and filtered using Picard version 1.85 (RRID:SCR_006525). Tumor samples with matched normal bile duct controls were analyzed using Strelka2.

### Somatic variant analysis

All exome samples were analyzed using VarScan2 (RRID:SCR_006849) and annotated using SNPEff (RRID:SCR_005191) to identify both somatic and germline variants. Default parameters were applied, except for a minimum variant allele frequency threshold of 0.01 (1% tumor variant allele frequency). The output variants were screened against COSMIC (Catalogue of Somatic Mutations in Cancer) and annotated against dbSNP. For the Cancer Hotspot Panel, identification of variants was performed using Ion Torrent Variant Caller software (hg19) and screened against COSMIC.

### Identification of potential actionable targets

OncoKB (RRID:SCR_014782), a precision oncology database developed and maintained at Memorial Sloan Kettering, was used to identify targets which may harbor actionable potential and are considered Level 4 (compelling biological evidence supports the biomarker as being predictive of response to an FDA approved or investigational drug). As such, there is no clinical trial evidence at present to support the use of a particular drug in this disease setting but the presence of the mutation serves as a rational candidate for further investigation. All identified genes are considered cancer genes by OncoKB™, based on their inclusion in the Sanger Cancer Gene Census, or Vogelstein et al.^11^.

### Mutational signatures

Mutational signatures were generated using the MutationalPatterns R package.

### Pathway analysis

Gene set enrichment analysis (GSEA, RRID:SCR_003199) was undertaken using the C2.cp.v7 gene set with genes ranked by their maximum SNV frequency using a bootstrapping approach to estimate the significance of a geneset enriched within the ranked set of features. This methodology randomly selects a set of genes of the same size as the tested geneset and calculates a normalised enrichment score (NES). This process is repeated for 1000 randomly sampled genesets. The resulting distribution of normalised enrichment scores is used to assign p-values, false discovery rate (FDR), and family-wise error rate (FWER) values for the given true enrichment score.

### Statistical analyses

Categorical variables were analysed using chi-squared and fisher’s exact tests with bonferroni correction for multiple testing. Non-parametric data was analysed using the Mann–Whitney U-test and survival analyses undertaken using Kaplan–Meier.

### Ethical approval

UK National Health Service Research Ethics Committee approval was obtained (UK REC 15/NW/0477) and the study was conducted according to the Declaration of Helsinki. All samples were obtained with informed consent.

## Results

### Clinical and pathological features

Forty-two patients were included for analysis (twenty five FFPE, seventeen fresh frozen samples). All patients were Caucasian, with a clinical diagnosis of idiopathic pCCA (Table 1). Patients with primary sclerosing cholangitis or chronic liver infection were excluded.

**Table 1:** Clinico-pathological characteristics of sequencing cohorts.

Clinicopathological characteristics		Exome (N = 25)	Hotspot (N = 17)
Gender	Male	14	11
	Female	11	6
Age (Median)		66 (IQR 18 years)	64 (IQR 16 years)
Resection	Hepatic and bile duct	19	14
	Bile duct only (Type II)	6	3
TNM (AJCC 2017)	I	1	1
	II	13	6
	IIIA	0	1
	IIIB	0	0
	IIIC	11	8
	IVA	0	1
	IVB	0	0
Radial margin	R1	8	3
	R0	17	14
Circumferential margin	R1	13	10
	R0	12	7
Peri-neural invasion	Yes	24	16
	No	1	1
Vascular invasion	Yes	15	9
	No	10	8
Cell differentiation	Well	4	1
	Moderate	15	10
	Poor	6	6
Precursor lesion	BiliN	0	0
	IPNB	1	0
	None	24	17
Risk factor	PSC	–	–
	Caroli’s disease	–	–
	Cholelithiasis/Choledocholiathiasis	4	1
	Choledochal Cyst	–	–
	Cirrhosis	–	–
	Hemochromatosis	–	–
	Chronic liver infection (HCV/HBV)	–	–
	Inflammatory Bowel Disease	–	–
	Chronic pancreatitis	–	–
	Type II Diabetes	–	1
	Non-alcoholic fatty liver disease	1	–
	Obesity	4	–
	Hypertension	7	2
	Excess alcohol consumption	1	–
	Cigarette smoking	3	2

Table summarizes the clinical and pathological characteristics of idiopathic PCCA patients included in this study. Histopathological staging is according to the AJCC 8th edition guidelines.

### Sequencing metrics

Median on-target mapping was 76% in the exome set and 100% in the Cancer Hotspot panel with sequencing depth of > 50 × and > 1000 × respectively. To examine the mutational landscape of the cohort, whole-exon coverage of 409 established cancer genes was interrogated in all 42 patients. High and low tumor mutational burden (TMB) are defined as ≥ 20 and ≤ 5 mutations per megabase/DNA respectively^12^. The median number of somatic mutations in the whole exome cohort was 13.57/MB (inter quartile range 9.12/MB, range 7.83–74.43), implying a moderate TMB.

### Most frequently mutated genes

Mutated genes converged into the seven oncogenic pathways as follows; RTK-RAS-PI3K (59.5%), p53 (54.8%), PI3K/mTOR (28.6%), NOTCH (14.3%), the cell cycle pathway (4.8%), the Wnt pathway (4.8%) and TGF-B pathway (4.8%). Further analysis demonstrated the most frequently mutated genes (including both SNV and Insertion/deletion) were (from most to least recurrent): *TP53, KRAS, mTOR, ABL1, NOTCH1, PBRM1, PIK3CA, NF1* and *EGFR.* Mutations within these genes are summarized in Table 2 with comparison of their mutational frequencies to published international datasets provided in Supplementary Table 1.

**Table 2:** Most frequent somatic mutations in the study population (n = 42).

Gene	Impact	Base change	Amino acid change	Mean allele frequency (%)	Variance	FATHMM pathogenic prediction Score	ClinVar significance	Candidate targeted therapeutics (OncoKB)
*ABL1*	Missense	c.748G > A	p.Gly250Arg	3.23	0.086	–	Likely pathogenic	Bosutinib Dasatinib Imatinib Nilotinib Ponatinib
	Missense	c.778G > A	p.Val260Met	3.62	0.212	–	Pathogenic	
*EGFR*	Structural	c.2602G > A	–	3.70	0	0.99	–	Afatinib Dacomitinib Erlotinib Gefitinib Osimertinib
	Missense	c.530C > T	p.Ser177Leu	4.60	0.0008	–	Uncertain	
*KRAS*	Structural	c.64C > A	p.Gln22Lys	2.00	0	0.99	Pathogenic	Sotorasib Cobimetinib Trametinib Binimetinib
	Missense	c.35G > A	p.Gly12Asp	4.39	0.0003	0.98	Pathogenic	
	Missense	c.35G > A	p.Gly12Asp	3.01	6.48	0.98	Pathogenic	
	Missense	c.183A > C	p.Gln61His	5.48	0	0.93	Pathogenic	
	Missense	c.35G > T	p.Gly12Val	4.39	0	0.98	Pathogenic	
	Missense	c.38G > A	p.Gly13Asp	4.56	0	0.98	Pathogenic	
*mTOR*	Missense	c.7438C > A	p.His2480Asn	1.35	0	–	–	Everolimus Temsirolimus
	Missense	c.3482G > A	p.Arg1161Gln	3.46	0.0001	0.99	Uncertain	
	Missense	c.94C > T	p.Arg32Trp	3.81	0.0004	0.99	–	
*NF1*	Frameshift	c.91_92delCA	p.His31fs	19.29	0.03	–	Pathogenic	Seleumetinib Trametinib Cobimetinib
	Frameshift	c.1882delT	p.Tyr628fs	24.50	0	–	Pathogenic	
	Frameshift	c.6852_6855delTTAC	p.Tyr2285fs	4.00	0	–	Pathogenic	
*NOTCH1*	Missense	c.5362G > A	p.Gly1788Ser	1.92	0.0001	0.98	Uncertain	–
	INDEL	c.4732_4734delGTG	p.Val1578del	2.17	1.06	–	–	
*PIK3CA*	Structural	c.3140A > T	p.His1047Leu	1.00	0	0.96	Pathogenic	Alpelisib and Fulvestrant
	Missense	c.3062A > G	p.Tyr1021Cys	16.80	0	1.00	Likely pathogenic	
	Missense	c.1624G > A	p.Glu542Lys	2.20	0	0.97	Pathogenic	
	Missense	c.3196G > A	p.Ala1066Thr	9.30	19.6	0.95	–	
*PBRM1*	Stop gain	c.2128C > T	p.Arg710*	2.49	0.0006	0.90	–	–
*TP53*	Missense	c.556G > A	p.Asp186Asn	1.00	0	0.99	Uncertain	–
	Frameshift	c.822dupC	p.Ser275fs	9.75	0.0054	–	–	

Somatic mutations with a tumor variant allele frequency ≥ 1% (and significantly < 50%) together with nucleotide and amino acid changes, FATHMM (functional analysis through hidden Markov models) pathogenic prediction score and National Library of Medicine ClinVar significance are summarized in table. FATHMM is a high throughout webserver capable of predicting functional consequences of coding and non-coding variants. Targeted therapeutics which may serve as candidates for further investigation are derived from the OncoKB database.

*TP53* (36%, 15/42 cases) was most frequently mutated, wherein frameshift mutation conferred a worse overall survival (HR 3.33, p < 0.033) (Supplemental Figure 2). This was followed by *KRAS* mutation (24%, 10/42 cases), which did not confer a significant survival difference.

A second tier of high frequency mutations were identified in oncogenes not typically associated with cholangiocarcinoma, including *mTOR* (17%, 7/42 cases) and *ABL1* (14%, 6/42 cases). Other oncogenes more often associated with intra-hepatic disease were observed at a higher frequency than published series including *PIK3CA* (12%, 5/42 cases) and *EGFR* (10%, 4/42 cases). The *NOTCH1* alterations (12%, 5/42 cases) are noteworthy as *NOTCH1* may be either tumor suppressive or oncogenic. Although not currently listed as targetable by OncoKB criteria, mutations within *SMO* and *KDR* oncogenes were also observed (7%, 3/42 cases respectively).

Known intra-hepatic cholangiocarcinoma-associated tumor suppressor genes including *NF1* and *PBRM1* (12%, 5/42 cases respectively) were also higher prevalence than published peri-hilar series. Often associated with extra-hepatic disease, *SMAD4* (5%, 2/42 cases) and *CDK2NA/CDK2NB* (2%, 1/42 cases) mutations were comparatively lower. Other low prevalence tumor suppressor genes included *KMT2D* (7%, 3/42 cases), *LRP1B* and *APC* (5%, 2/42 cases respectively).

Lower prevalence mutations pertaining to cell cycle, growth and proliferation included *AKT1*, *CDH1*, *CTNNB1*, *FGFR1*, *FGFR2*, *JAK2*, *MPL*, *NPM1* and *RB1*. Somatic mutations in DNA damage repair genes were low frequency and included *ATM*, *CHEK1*, *BRCA1* and *BRCA2*.

Pertinent genes which did not demonstrate mutation at a ≥ 1% allele frequency included *IDH1/2*, *PTEN*, *NRAS*, *BRAF* and *BAP1*.

Applying OncoKB criteria, twenty-five of forty-two patients had at least one mutation within an established cancer gene which may have future actionable potential. At least two mutations were observed in nine patients with *ABL1* co-occurring with *KRAS* (5%, 2/42 cases) and *PI3KCA* (7%, 3/42 cases), whilst mutations in *EGFR* were shared with *mTOR* (5%, 2/42 cases). Of these nine patients, *TP53* mutation was present in two cases, each of which harbored both a *KRAS* and *ATM* mutation.

### Cluster analysis

Principle component analysis (PCA) using a sparse approach was conducted on non-transformed whole exome sequencing data. Following removal of 2 outliers on initial PCA, all remaining patients clustered closely together on the basis of SNV containing exons (Supplementary Figure 1).

### Hyper-mutated phenotype

In patients with ≥ 10 somatic mutations/Mb of DNA, mutations in chromatin remodeling ATPase *SMARCA4* occurred most frequently, being present in four hyper-mutated cases (mean 55.4 mutations/Mb).

Genes with the highest mean mutational burden included the somatostatin receptor *SSTR3* (278.1/Mb), the mitogen-activated-protein-kinase *MAPK1* (247.2/Mb) and *KRAS* (214.8/Mb), each present in three hypermutated cases.

Mismatch repair mutations were low prevalence with *MSH2* mutation observed in one patient. Mutations in other classical mismatch repairs genes were absent.

### Mutational signatures

Somatic SNV signatures were compared between paired tumors and normal bile ducts (V3 COSMIC SBS signatures)^13^. Strength of association in tumors was consistently higher than paired normal bile duct in signature 2 (activity of APOBEC family of cytidine deaminases), signature 3 (defective homologous recombination DNA damage repair) and signature 4 (tobacco smoking).

### Germline mutations

Paired normal bile ducts were assessed for mutations associated with known familial cancer syndromes (Supplementary Table 2). Missense mutations of uncertain or conflicting clinical significance were found in seven patients with *APC, ATM, BRCA2, MEN1, POLD1, RET* and *TSC1* each present in at least one case. Uncertain mutations were more frequent in *BRCA1* (4 cases) and *ATM* (2 cases). None of these patients described previous personal history of cancer or known first-degree relatives with familial cancer syndromes.

### Somatic mutations not previously associated with cholangiocarcinoma

**Figure 1:**
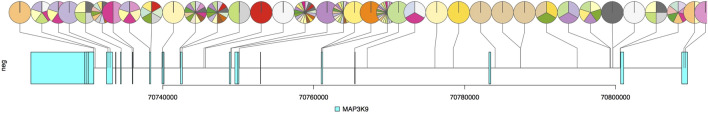
Genomic locations of SNVs in *MAP3K9*. Figure 1 demonstrates the large number and locations of SNVs across the *MAP3K9* gene. Exons are depicted as blue blocks. Thirty-seven unique SNVs were observed in total. Each pie chart portrays the number of patients who were found to have that specific variant at that genomic location. The color of each pie chart slice is specific to an individual patient and is conserved across all pie charts.

Whole exome sequencing identified numerous non-silent mutations in *MAP3K9* (Mitogen Activated Protein Kinase 9) (Fig. 1), which is not associated with any cholangiocarcinoma subtype. A CCT deletion (p.Glu38del) was present at a somatic allele frequency in 10 of the 25 whole exome sequenced tumors (mean allele frequency 13.72%, variance 0.005) and was associated with the presence of peri-vascular tumor invasion (p < 0.018). This specific mutation was present independent of both *BRAF* and *NRAS* mutations and contained within a region of known structural gain^14^, and was present at a germline frequency in a further 12 tumors (mean allele frequency of 43.8%, variance 0.09). In those tumors with the germline variant, it was also evident in matched normal bile duct samples at the same allele frequency. A separate somatic *MAP3K9* frameshift (p.Ser327fs) was also present in one additional tumor.

### Gene set enrichment analysis

GSEA revealed 795 SNV-enriched pathways within tumor tissue. Twenty-one cancer-related pathways achieved significance (false discovery rate < 5%) and are summarized in descending order in Table 3 (SNVs are detailed in Supplementary Table 3).

**Table 3:** Single nucleotide enriched cancer and immune response pathways.

Pathway	Role	FDR	FWER	P value (one-tail test)	Total SNV altered genes within pathway
Dectin-2	Antigen uptake for T-cell presentation Toll-like receptor independent production of cytokines	0.0010	0.0080	< 0.001	20
Termination of O-Glycan Biosynthesis	Post-translational modification Leucocyte trafficking Mucin glycoslyation	0.0030	0.0190	< 0.001	19
PD-1/PD-L1	Down-regulation of T-cell response	0.0070	0.0900	< 0.001	17
Translocation of ZAP-70	T-cell polarization	0.0095	0.1260	< 0.001	17
Phosphorylation of CD3 and TCR zeta chains	T-Cell receptor signal transduction	0.0098	0.1490	< 0.001	17
MET activates PTK2	Hepatocyte growth factor (HGF)-induced cell migration	0.0100	0.1770	< 0.001	27
Collagen degradation	Tumor invasion	0.0152	0.2790	< 0.001	44
Collagen biosynthesis	Tumor invasion	0.0174	0.3500	< 0.001	45
NCAM1 (CD56)	Cell–Cell adhesion Neurite outgrowth Immune surveillance (Helper T-cell expansion)	0.0190	0.4160	< 0.001	28
Class I MHC folding	Antigen presentation	0.0210	0.5080	< 0.001	22
TH1TH2	Regulation of Helper T-cell response	0.0220	0.4640	< 0.001	9
NOTCH HLH Transcription	Regulation of embryonic and adult cell differentiation	0.0297	0.7420	< 0.001	19
NRAGE signals death through JNK	Cell death signaling (apoptosis)	0.0390	0.8680	< 0.001	36
Receptor type tyrosine protein phosphatase	Regulation of synaptic organization	0.0450	0.9010	< 0.006	16
Intestinal immune network	Intestinal IgA production	0.0460	0.8990	< 0.001	29
Interferon gamma	Cytokine signaling	0.0460	0.9340	< 0.001	52
ARF6 trafficking	MET receptor signal transduction Vesicle mediated cell transport Intra-cellular recycling of PD-L1	0.0470	0.9210	< 0.001	38
SEMA4D induced migration	Cell migration and neuronal growth cone collapse B-cell and dendritic cell activation via CD72 binding	0.0470	0.9200	< 0.004	14
PITX2	Transcriptional regulation in cell proliferation and morphogenesis	0.0480	0.9420	< 0.009	8
RHO GTPases activate ROCKs	RHO GTPase signal transduction Regulation of cell mobility, plasticity and migration Regulation of tumor associated fibroblast migration	0.0490	0.9530	< 0.004	14
Mismatch repair	DNA repair	0.0490	0.9530	< 0.016	9

Table outlines significant single nucleotide variant enriched pathways (false discovery rate < 5%) identified from GSEA that pertain to cancer and immune biology. Pathways are listed in descending order of significance and highlight the propensity toward host immune pathways. A description of the biological role and number of genes containing altered single nucleotide variants is included.

The most enriched pathways pertain to host immune response, with the innate dendritic cell associated C-type lectin-2 (Dectin-2) family (FDR 0.001, p < 0.001) and O-Glycan biosynthesis (FDR 0.003, p < 0.001) achieving greatest significance. Both pathways have critical roles in metastatic formation, whereby Kupffer cells in the liver engulf metastatic cancer cells in a Dectin-2-dependent manner^15^, and O-Glycans facilitate new tissue invasion^16^.

**Figure 2:**
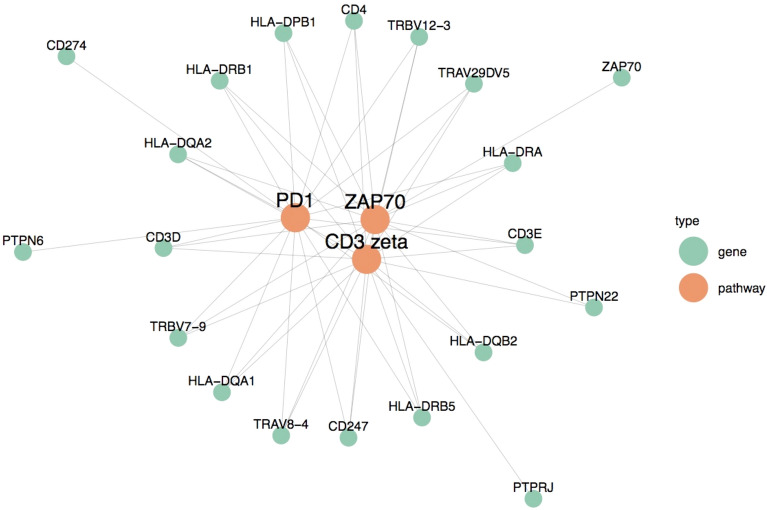
T-cell receptor signaling. This network diagram outlines the overlap of genes shared between critical TCR pathways including PD-1, CD3 phosphorylation and ZAP70 translocation. Edges connect SNV enriched genes to the pathways they are involved in, and show all genes that are involved in all three pathways. *HLA-DRB1, HLA-DRA* and *HLA-DRB5* are shared by all three of these pathways.

Programmed cell death protein 1 (PD-1) was the most mutated adaptive response (FDR 0.007) and is variably expressed in cholangiocarcinoma^17,18^. Other highly significant adaptive pathways relate to T-cell receptor-induced activation of cytotoxic T-cells and have not been described in cholangiocarcinoma including cluster of differentiation 3 (CD3) phosphorylation (FDR 0.009), translocation of zeta-chain associated protein kinase 70 (ZAP70) to the immunological synapse (FDR 0.039) and the TH1TH2 pathway (FDR 0.02). Figure 2 outlines overlapping genes in these pathways, with aberrant *HLA-DRB1, HLA-DRA* and *HLA-DRB5* shared by all four. The human leucocyte antigen-DR isotype (HLA-DR) is a major histocompatibility complex (MHC) Class II cell surface receptor, which presents extra-cellular peptides to Helper T-cells^19^.

**Figure 3:**
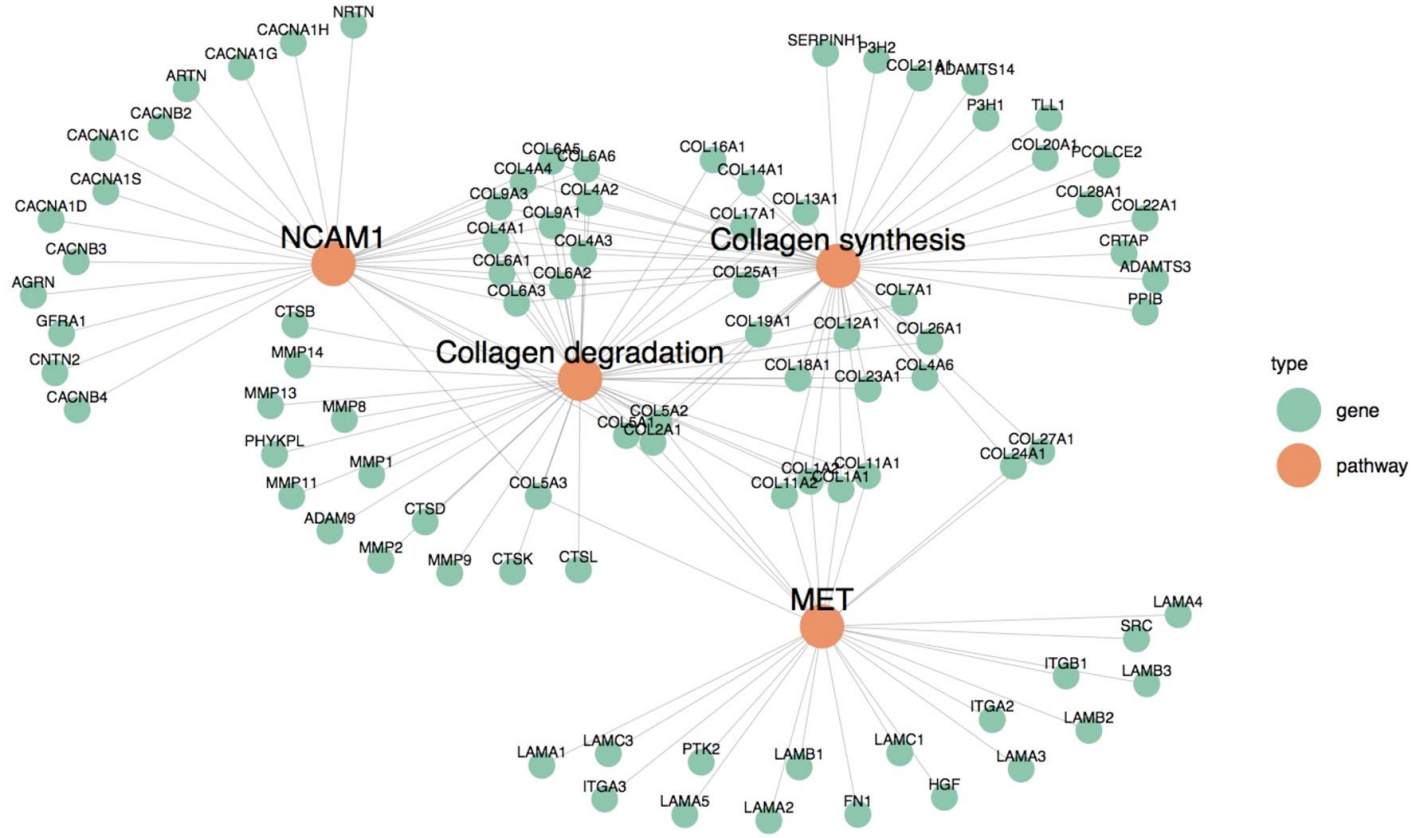
Cell proliferation pathways pertaining to collagen synthesis and degradation. This network diagram highlights the overlap of genes in the oncogenic pathways NCAM1 and MET. Edges connect SNV enriched genes to the pathways they are involved in, and show a central group of genes involved in multiple pathways converge into the collagen synthesis and degradation pathways. Mutations within *COL5A1, COL5A2, COL5A3* and *COL2A1* are shared by all four pathways.

Tyrosine-protein kinase Met (MET) activation of protein tyrosine kinase 2 (PTK2) (FDR 0.01) was the most altered cell migration/proliferation pathway. Although the role of MET signaling in cholangiocarcinogenesis is unknown, overexpression of c-MET is a poor prognostic feature in intra and extra-hepatic disease^20^. This pathway substantially overlaps Neural Cell Adhesion Molecule 1 (NCAM1) (FDR 0.019), which positively correlates with peri-neural invasion across biliary cancers^21^. Overlapping genes converged with collagen synthesis and degradation, with collagen type V subtypes, *COL5A1, COL5A2, COL5A3* and type II alpha 1 (*COL2A1*) common to all four pathways (Fig. 3).

Components of other well-characterized oncogenic pathways in cholangiocarcinoma achieved significance, including developmental NOTCH signaling (FDR 0.0279). A number of highly mutated cell signaling pathways are new to cholangiocarcinoma and possess immune-based functions including ADP-ribosylation factor 6 (ARF6) trafficking (FDR 0.047), Semaphorin 4D (SEMA4D) (FDR 0.047) and Rho-Roc signaling (FDR 0.049).

## Discussion

This study describes the first exploratory whole exome sequencing analysis of a Western idiopathic peri-hilar cholangiocarcinoma cohort. We identified driver mutations distinct from Asian peri-hilar whole exome studies and from targeted sequencing series that have included Western patients (Supplementary Table 1). Mutated genes range from those not typically associated with pCCA*,* to those entirely new to biliary cancer such as *MAP3K9*. Somatic mutations within at least one well established cancer gene (OncoKB level 4) were observed in 60% of cases. These mutations necessitate further functional analysis, however their presence suggests a significant proportion of Western idiopathic peri-hilar patients may be eligible for contemporary basket trials of target based therapies.

Mutations in *mTOR* and *ABL1* are noteworthy given they are activating in other cancers resulting in increased cell proliferation^22,23^, and may contribute to cholangiocarcinogenesis in this fashion. Whilst components of the mTOR pathway have been identified in extra-hepatic cholangiocarcinoma^24^, together with enriched mTOR signaling at the transcriptome level^25^, mutations specifically within *mTOR* have been absent. The ABL kinases are a well-known driver of leukemia, yet are increasingly implicated in the progression of several solid cancers independent of their fusion oncoproteins^26^. Both of these oncogenes may therefore serve as peri-hilar biomarkers to indicate benefit from new and existing targeted therapies. This encompasses historically efficacious therapeutics in unselected advanced cholangiocarcinoma such as the mTOR inhibitor Everolimus^27^, now included in new Phase II solid cancer trials (NCT04591431).

More typically intra-hepatic, *PIK3CA* and *EGFR* are closely related to *mTOR* and *ABL1* respectively^28^. *EGFR* mutations in particular, suggest continuing investigation into oral tyrosine kinase inhibitors in pCCA is justified despite limited efficacies in unselected advanced disease^29^. Other less frequent oncogenes such as *SMO,* broaden eligibility for unexplored therapeutics such as Vismodegib (NCT02091141).

We identified higher frequency *NOTCH1* mutations than published series. Although NOTCH1 overexpression is associated with poor differentiation in extra-hepatic cholangiocarcinoma^30^, NOTCH signaling may be oncogenic or tumor suppressive^31^. The activation status of the alterations identified here is unknown. A trial of the NOTCH inhibitor LY3039478 in advanced solid cancers is ongoing (NCT02784795). If these mutations are activating, significant proportions of idiopathic peri-hilar patients are eligible. Other suppressors overlap iCCA, including *TP53*, *PBRM1* and *NF1*, yet deviate from published extra-hepatic series with low frequency *SMAD4* and *CDK2NA/B* aberrations (Supplemental Table 1). The association between *TP53* frameshift mutation and poor prognosis is notable given this has been well described in iCCA as opposed to pCCA^32^. *KMT2D* mutations are also noteworthy*,* given their primary association with non-Hodgkin lymphoma^33^, and vulnerability to glycolytic inhibitors^34^.

Our dataset also expands on the limited knowledgebase of cancer predisposing germline mutations in cholangiocarcinoma. We identified alterations of uncertain significance within DNA damage repair (DDR) genes including *BRCA1*, *BRCA2* and *ATM*, which were pathogenic in two recent U.S. studies that included twenty-one and fifty-three extra-hepatic cases respectively^35,36^. Together with the homologous somatic DDR signature found in our series, our data lends further support for DDR testing in idiopathic pCCA. This may be clinically significant given the efficacy of Olaparib in germline *BRCA* mutated metastatic pancreatic adenocarcinoma^37^.

WES demonstrated the majority of tumors clustered closely together and identified novel coding mutations in *MAP3K9*. Somatic mutations in *MAP3K9* are implicated in retroperitoneal neuroblastoma^38^, and esophageal carcinogenesis^39^. The p.Glu38del in *MAP3K9* is noteworthy given its association with increased peri-vascular tumor invasion in our series. MAP3K9 activates MEK/ERK independently of RAF^40^, and may contribute to cholangiocarcinogenesis by activating the downstream targets of ERK. With high prevalence at somatic and germline allele frequencies, the potential driver role of MAP3K9 in idiopathic pCCA warrants clarity given mutations are gain of function in lung cancer^41^, and loss of function in melanoma wherein its attenuation may lead to chemo-resistance^42^. If mutation is loss of function, reactivation of downstream signaling may be advantageous. Conversely, if activating, *MAP3K9* inhibition may lead to cancer cell suppression as observed in pancreatic cancer models^43^, and thus represents a novel target of significance.

Regarding tumor immunology, the median TMB > 10 mutations/MB of DNA is higher than published datasets^44^. Although the mismatch repair pathway achieved significance, classical mismatch repair mutations were absent. TMB correlates well with neo-epitope production, thus this TMB is of potential clinical utility given > 10 mutations/MB cutoffs infer benefit from immunotherapy^45^. Responses to immune checkpoint inhibition in unselected advanced cholangiocarcinoma have been poor, suggesting a low T-cell infiltrated microenvironment^10^. Interestingly, our gene set enrichment analysis demonstrates altered tumor T-cell receptor signaling with substantial overlap in HLA genes. Given increased HLA expression is associated with higher immune infiltration^46^, aberrant regulation of these HLA genes may facilitate immune evasion and provides new avenues for exploring the limitations of immunotherapy in pCCA.

The majority of the mutation-enriched pathways are new to cholangiocarcinoma. We did not seek to mechanistically evaluate these, rather, we sought to open new avenues for functional investigation. New immune pathways range from those that may facilitate metastatic formation such as Dectin-2 and O-Glycan biosynthesis, to those, which impact checkpoints, such as ARF6, critical for PD-L1 dynamics in pancreatic cancer^47^. Other oncogenic pathways included those known to contribute to poor prognosis in cholangiocarcinoma yet which are poorly defined in terms of their components, such as MET and NCAM1.

As a sequencing tool, WES provides summative measures of mutations within the bulk tumor tissue and cannot differentiate relative contributions of each stromal component. Single cell RNA-sequencing is a powerful new modality for unravelling intra-tumoral heterogeneity, which has identified demonstrable differences in single cell gene expression between defined subsets of malignant epithelial and immune cells in both iCCA and dCCA^48,49^. Future studies should address single cell gene expression in a pCCA specific context.

In summary, this descriptive study contributes a significant advance to the idiopathic pCCA knowledge base. Validation of these mutations in larger cohorts is essential, as is as their functional analysis in the laboratory. Our data supports clinical sequencing of cholangiocarcinoma, as despite some overlap with iCCA, there remains an unmet need for peri-hilar specific biomarkers. Umbrella and basket trials including the mutated cancer genes observed in our series are needed to bring this subtype into line with progress made in iCCA.

## Supplementary Information

Supplementary Table 1.

Supplementary Table 2.

Supplementary Table 3.

Supplementary Figure 1.

Supplementary Figure 2.

Supplementary Legends.

## Data Availability

Raw data for this study were generated at the Liverpool Centre for Genomic Research (University of Liverpool, U.K) and are available at the European Nucleotide Archive (Study ID PRJEB59167) (ERP144229) (https://www.ebi.ac.uk/ena/browser/view/PRJEB59167).

## Abbreviations

AACR: American Association of Cancer Research
ABL1: Tyrosine protein kinase ABL1 (Abelson 1)
AJCC: American Joint Committee on Cancer Classification
AKT: Protein Kinase B
APC: Adenomatous polyposis coli
APOBEC: Apolipoprotein B mRNA-editing enzyme catalytic polypeptide
ARID1A: AT-rich interactive domain-containing protein 1A
ARF6: ADP (adenosine diphosphate)-ribosylation factor 6
ATM: ATM (ataxia-telangiectasia) serine/threonine kinase
BAP1: BRCA-associated protein 1
BiliN: Biliary intra-epithelial neoplasia
BRAF: B-Raf proto-oncogene
BRCA1: Breast cancer gene 1
BRCA2: Breast cancer gene 2
BTC: Biliary tract cancer
CDH1: Cadherin-1
CD3: Cluster of differentiation 3
CDK2NA: Cyclin dependent kinase inhibitor 2A
CDK2NB: Cyclin dependent kinase inhibitor 2B
CKD4: Cyclin dependent 4
COL2A1: Collagen type II alpha 1 chain
COL5A1: Collagen type V alpha 1 chain
COL5A2: Collagen type V alpha 2 chain
COL5A3: Collagen type V alpha 3 chain
COSMIC: Catalogue of somatic mutations in cancer
CTNNB1: Catenin beta-1
dCCA: Distal cholangiocarcinoma
dbSNP: Single nucleotide polymorphism database
DNA: Deoxyribonucleic acid
DDR: DNA damage repair
eCCA: Extra-hepatic cholangiocarcinoma
EGFR: Epidermal growth factor
ERBB2/Her2: Erythroblastic oncogene B/Human epidermal growth factor receptor 2
ERK: Extracellular signal-regulated kinases
FATHMM: Functional analysis through hidden Markov models
FDA: U.S. Food and Drug Administration
FDR: False discovery rate
FFPE: Formalin fixed paraffin embedded
FGFR1: Fibroblast growth factor 1
FGFR2: Fibroblast growth factor 2
FWER: Family wise error rate
GSEA: Gene set enrichment analysis
HBV: Hepatitis B virus
HCV: Hepatitis C virus
HDGF: Hepatoma-derived-growth-factor
HLA: Human leukocyte antigen
HLA-DR: Human leukocyte antigen DR isotype
HLA-DRA: Human leukocyte antigen DR alpha chain
HLA-DRB1: Human leukocyte antigen DR beta 1 chain
HLA-DRB5: Human leukocyte antigen DR beta 5 chain
HR: Hazard ratio
iCCA: Intra-hepatic cholangiocarcinoma
IDH1: Isocitrate dehydrogenase 1
IDH2: Isocitrate dehydrogenase 2
IPNB: Intra-ductal papillary neoplasm of the bile duct
JAK2: Janus kinase 2
JNK: C-Jun N-terminal kinases
KDR: Kinase insert domain receptor
KMT2D: Histone-lysine N-methyltransferase 2D
KRAS: Kirsten rat sarcoma
LRP1B: Low-density lipoprotein receptor related protein 1B
MAPK: Mitogen-activated protein kinase
MAPK1: Mitogen-activated protein kinase 1
MAP3K9: Mitogen-activated protein kinase 9
MDM2: Mouse double minute 2 homolog
MEK: Mitogen-activated protein kinase kinase
MEN1: Multiple endocrine neoplasia type 1
MET: Tyrosine-protein kinase met
MHC: Major histocompatibility complex
MPL: Myeloproliferative leukaemia protein (Thrombopoietin receptor)
MSH2: MutS homolog 2
mTOR: Mechanistic target of rapamycin
MUTYH: MutY DNA glycosylase
NCAM1: Neural cell adhesion molecule 1
NCT: National clinical trial
NES: Normalized enrichment score
NF1: Neurofibromin 1
NOTCH1: Notch homologue 1
NPM1: Nucleophosmin
NRAGE: Melanoma-associated antigen D1
NRAS: Neuroblastoma RAS
PBRM1: Polybromo 1
PCA: Principle component analysis
pCCA: Peri-hilar cholangiocarcinoma
PD-1: Programmed cell death 1 protein
PDL-1: Programmed cell death ligand 1
PIK3CA: Phosphatidylinositol-4,5-bisphosphate 3-kinase catalytic subunit alpha
PITX2: Paired-like homeodomain transcription factor 2
POLD1: DNA polymerase delta 1
PSC: Primary sclerosing cholangitis
PTEN: Phosphatase and tensin homolog
RAF: Raf-1 proto-oncogene
RB1: Retinoblastoma transcriptional corepressor 1
RET: RET (rearranged during transfection) proto-oncogene
RHO: Rho GTPase
ROC: Rho-associated protein kinase
SEMA4D: Semaphorin-4D
SMAD4: Mothers against decapentaplegic homolog 4
SMARCA4: Transcription activator BRG1
SMO: Smoothened
SNV: Single Nucleotide Variant
SSTR3: Somatostatin receptor type 3
STK11: Serine Threonine Kinase 11
TCR: T-cell receptor
Th1Th2: T-helper 1/T-helper 2
TMB: Tumour mutational burden
TP53: Tumour suppressor protein 53
TSC1: TSC (tuberous sclerosis 1) complex subunit 1
UBR4: Ubiquitin protein ligase E3 component n-recognin 4
WES: Whole exome sequencing
WNT: Wingless-related integration site
ZAP 70: Zeta-chain associated protein kinase 70

## References

[1] Banales JM, Marin JJG, Lamarca A, Rodrigues PM, Khan SA, Roberts LR (2020). Cholangiocarcinoma 2020: The next horizon in mechanisms and management. Nat. Rev. Gastroenterol. Hepatol..

[2] Bertuccio P, Malvezzi M, Carioli G, Hashim D, Boffetta P, El-Serag HB (2019). Global trends in mortality from intra-hepatic and extra-hepatic cholangiocarcinoma. J. Hepatol..

[3] Shin HR, Oh JK, Masuyer E, Curado MP, Bouvard V, Fang YY (2010). Epidemiology of cholangiocarcinoma: An update focusing on risk factors. Cancer Sci..

[4] DeOliveira ML, Cunningham SC, Cameron JL, Kamangar F, Winter JM, Lillemoe KD (2007). Cholangiocarcinoma: Thirty-one-year experience with 564 patients at a single institution. Ann. Surg..

[5] Nakamura H, Arai Y, Totoki Y, Shirota T, Elzawahry A, Kato M (2015). Genomic sprectra of biliary tract cancer. Nat. Genet..

[6] Abou-Alfa GK, Macarulla T, Javle MM, Kelley RK, Lubner SJ, Adeva J (2020). Ivosidenib in IDH1-mutant, chemotherapy-refractory cholangiocarcinoma (ClarIDHy): A multicentre, randomised, double-blind, placebo-controlled, phase 3 study. Lancet Oncol..

[7] Abou-Alfa GK, Sahai V, Hollebecque A, Vaccaro G, Melisi D, Al-Rajabi R (2020). Pemigatinib for previously treated, locally advanced or metastatic cholangiocarcinoma: A multicentre, open-label, phase 2 study. Lancet Oncol..

[8] Jusakul A, Cutcutache I, Yong CH, Lim JQ, Huang MN, Padmanabhan N (2017). Whole genome and epigenomic landscapes of etiologically distinct subtypes of cholangiocarcinoma Cancer. Discovery.

[9] Wardell CP, Fujita M, Yamada T, Simbolo M, Fassan M, Karlic R (2018). Genomic characterization of biliary tract cancer identified driver genes and predisposing mutations. J. Hepatol..

[10] Piha-Paul SA, Oh DY, Ueno M, Malka D, Chung HC, Nagrial A (2020). Efficacy and safety of pembrolizumab for the treatment of advanced biliary cancer: Results from the KEYNOTE-158 and KEYNOTE-028 studies. Int. J. Cancer.

[11] Vogelstein B, Papadopoulos N, Velculescu VE, Zhou S, Diaz LA, Kinzler KW (2013). Cancer genome landscapes. Science.

[12] Alexandrov LB, Nik-Zainal S, Wedge DC, Aparicio SA, Behjati S, Biankin AV (2013). Signatures of mutational processed in human cancer. Nature.

[13] Alexandrov LB, Kim J, Haradhvala NJ, Huang MN, Tian Ng AW, Wu Y (2020). The repertoire of mutational signatures in human cancer. Nature.

[14] Coe BP, Witherspoon K, Rosenfeld JA, van Bon BW, Vulto-van Silfhout AT, Bosco P (2014). Refining analyses of copy number variation identifies specific genes associated with developmental delay. Nat. Genet..

[15] Kimura Y, Inoue A, Hangai S, Saijo S, Negishi H, Nishio J (2016). The innate immune receptor Dectin-2 mediates phagocytosis of cancer cells by Kupffer cells for the suppression of liver metastasis. Proc. Natl. Acad. Sci. U.S.A..

[16] Varki A (2015). Essentials of glycobiology.

[17] Walter D, Herrmann E, Schnitzbauer AA, Zeuzem S, Hansmann ML, Peveling-Oberhag J (2017). PD-L1 expression in extra-hepatic cholangiocarcinoma. Histopathology.

[18] Ma K, Wei X, Dong D, Wu Y, Geng Q, Li E (2017). PD-L1 and PD-1 expression correlate with prognosis in extra-hepatic cholangiocarcinoma. Oncol. Lett..

[19] Baecher-Allan C, Wolf E, Hafler DA (2006). MHC class II expression identifies functionally distinct human regulatory T cells. J. Immunol..

[20] Miyamoto M, Ojima H, Iwasaki M, Shimizu H, Kokubu A, Hiraoka N (2011). Prognostic significance of overexpression of c-Met oncoprotein in cholangiocarcinoma. Br. J. Cancer.

[21] Seki H, Tanaka J, Sato Y, Kato Y, Umezawa A, Koyama K (1993). Neural cell adhesion molecule (NCAM) and perineural invasion in bile duct cancer. J. Surg. Oncol..

[22] Grabiner BC, Nardi V, Birsoy K, Possemato R, Shen K, Sinha S (2014). A diverse array of cancer-associated MTOR mutations are hyperactivating and can predict rapamycin sensitivity. Cancer Discov..

[23] Greuber EK, Smith-Pearson P, Wang J, Pendergast AM (2013). Role of ABL family kinases in cancer: From leukaemia to solid tumours. Nat. Rev. Cancer.

[24] Churi CR, Shroff R, Wang Y, Rashid A, Kang HC, Weatherly J (2014). Mutation profling in cholangiocarcinoma: Prognostic and therapeutic implications. PLoS ONE.

[25] Montal R, Sia D, Montironi C, Leow WQ, Esteban-Fabró R, Pinyol R (2020). Molecular classification and therapeutic targets in extra-hepatic cholangiocarcinoma. J. Hepatol..

[26] Wang J, Pendergast AM (2015). The emerging role of ABL kinases in solid tumors. Trends Cancer.

[27] Lau DK, Tay RY, Yeung YH, Chionh F, Mooi J, Murone C (2018). Phase II study of everolimus (RAD001) monotherapy as first-line treatment in advanced biliary tract cancer with biomarker exploration: The RADiChol Study. Br. J. Cancer.

[28] Falcomata C, Barthel S, Ulrich A, Diersch S, Veltkamp C, Rad L (2021). Genetic screens identify a context-specific PI3K/p27Kip1 node driving extrahepatic biliary cancer. Cancer Discov..

[29] Moehler M, Maderer A, Ehrlich A, Foerster F, Schad A, Nickolay T (2019). Safety and efficacy of afatinib as add-on to standard therapy of gemcitabine/cisplatin in chemotherapy-naive patients with advanced biliary tract cancer: An open-label, phase I trial with an extensive biomarker program. BMC Cancer.

[30] Aoki S, Mizuma M, Takahashi Y, Haji Y, Okada R, Abe T (2016). Aberrant activation of Notch signaling in extrahepatic cholangiocarcinoma: Clinicopathological features and therapeutic potential for cancer stem cell-like properties. BMC Cancer.

[31] Parmigiani E, Taylor V, Giachino C (2020). Oncogenic and tumor-suppressive functions of NOTCH signaling in glioma. Cells.

[32] Simbolo M, Vicentini C, Ruzzenente A, Brunelli M, Conci S, Fassan M (2018). Genetic alterations analysis in prognostic stratified groups identified TP53 and ARID1A as poor clinical performance markers in intrahepatic cholangiocarcinoma. Sci. Rep..

[33] Morin RD, Mendez-Lago M, Mungall AJ, Goya R, Mungall KL, Corbett RD (2011). Frequent mutation of histone-modifying genes in non-Hodgkin lymphoma. Nature.

[34] Alam H, Tang M, Maitituoheti M, Dhar SS, Kumar M, Han CY (2020). KMT2D deficiency impairs super-enhancers to confer a glycolytic vulnerability in lung cancer. Cancer Cell.

[35] Maynard H, Stadler ZK, Berger MF, Solit DB, Ly M, Lowery MA (2020). Germline alterations in patients with biliary tract cancers: A spectrum of significant and previously underappreciated findings. Cancer.

[36] Uson Junior PL, Kunze KL, Golafshar MA, Riegert-Johnson D, Boardman L, Borad MJ (2022). Germline cancer susceptibility gene testing in unselected patients with hepatobiliary cancers: A multi-center prospective study. Cancer Prev. Res..

[37] Golan T, Hammel P, Reni M, Van Cutsem E, Macarulla T, Hall MJ (2019). Maintenance olaparib for germline *BRCA*-mutated metastatic pancreatic cancer. N Engl J Med.

[38] Tivnan A, Tracey L, Buckley PG, Alcock LC, Davidoff AM, Stallings RL (2011). MicroRNA-34a is a potent suppressor molecule in vivo in neuroblastoma. BMC Cancer.

[39] Chen J, Guo L, Peiffer DA, Zhou L, Chan OT, Bibikova M (2008). Genomic profiling of 766 cancer related genes in archived esophageal normal and carcinoma tissues. Int. J. Cancer.

[40] Durkin JT, Holskin BP, Kopec KK, Reed MS, Spais CM, Steffy BM (2004). Phosphoregulation of mixed-lineage kinase 1 activity by multiple phosphorylation in the activation loop. Biochemistry.

[41] Fawdar S, Trotter EW, Li Y, Stephenson NL, Hanke F, Marusiak AA (2013). Targeted genetic dependency screen facilitates identification of actionable mutations in FGFR4, MAP3K9, and PAK5 in lung cancer. Proc. Natl. Acad. Sci. U.S.A..

[42] Stark MS, Woods SL, Gartside MG, Bonazzi VF, Dutton-Regester K, Aoude LG (2011). Frequent somatic mutations in MAP3K5 and MAP3K9 in metastatic melanoma identified by exome sequencing. Nat. Genet..

[43] Xia J, Cao T, Ma C, Shi Y, Sun Y, Wang ZP (2018). miR-7 suppresses tumor progression by directly targeting MAP3K9 in pancreatic cancer. Mol. Ther. Nucleic Acids..

[44] Weinberg BA, Xiu J, Lindberg MR, Shields AF, Hwang JJ, Poorman K (2019). Molecular profiling of biliary cancers reveals distinct molecular alterations and potential therapeutic targets. J. Gastrointest. Oncol..

[45] Chan TA, Yarchoan M, Jaffee E, Swanton C, Quezada SA, Stenzinger A (2019). Development of tumor mutation burden as an immunotherapy biomarker: Utility for the oncology clinic. Ann. Oncol..

[46] Schaafsma E, Fugle CM, Wang X, Cheng C (2021). Pan-cancer association of HLA gene expression with cancer prognosis and immunotherapy efficacy. Br. J. Cancer.

[47] Hashimoto S, Furukawa S, Hashimoto A, Tsutaho A, Fukao A, Sakamura Y (2019). ARF6 and AMAP1 are major targets of KRAS and TP53 mutations to promote invasion, PD-L1 dynamics and immune evasion of pancreatic cancer. Proc. Natl. Acad. Sci. U.S.A..

[48] Zhang M, Yang H, Wan L, Wang Z, Wang H, Ge C (2020). Single-cell transcriptomic architecture and intercellular crosstalk of human intrahepatic cholangiocarcinoma. J. Hepatol..

[49] Li H, Qu L, Yang Y, Zhang H, Li X, Zhang X (2022). Single-cell transcriptomic architecture unraveling the complexity of tumor heterogeneity in distal cholangiocarcinoma. Cell Mol. Gastroenterol. Hepatol..

