# Supplementary material for: Genomic profiling of idiopathic peri-hilar cholangiocarcinoma reveals new targets and mutational pathways
Source: Sci Rep. 2023 Apr 24;13:6681. doi: 10.1038/s41598-023-33096-0 (PMC10126102; doi:10.1038/s41598-023-33096-0)
Supplement: Supplementary file 1 — Supplementary Table 1. [file 41598_2023_33096_MOESM1_ESM.pdf]

Supplementary Table 1 – Comparison of most frequently mutated genes in Liverpool cohort with published international peri-hilar and extra-hepatic series. Supplementary table 1 provides a comparison of somatic mutations observed in the Liverpool series with international published peri-hilar datasets. These are reported as percentages (with number of cases given in brackets). In eight of these studies, peri-hilar cholangiocarcinoma is considered under the umbrella term of 'extra-hepatic cholangiocarcinoma' and the total number of mutations reported therefore reflects combined peri-hilar and distal cholangiocarcinoma cases (the number of peri-hilar tumors is given is this has been explicitly stated by the authors). This table illustrates the low numbers of pCCA that have been sequenced globally and in Western cohorts in particular.

* = Study included liver fluke positive disease.

** = Number of mutated cases for the named gene was not described according to subtype. The given percentage is representative of the total number of patients analyzed in that particular study including intra-hepatic, peri-hilar, and distal cholangiocarcinoma cases, and gallbladder cancers and thus percentage only is described herein.

*** = Whole exome sequencing in each of these studies was undertaken on Japanese samples only. The total number thus represents whole exome and targeted sequencing with the number in brackets indicating the number of samples that underwent whole exome sequencing in Japan.

**Quinn et al.**	**2021**	**UK**	**Whole exome Targeted**	**42**	**-**	**36% (15)**	**24% (10)**	**17% (7)**	**14% (6)**	**12% (5)**	**12% (5)**	**12% (5)**	**12% (5)**	**10% (4)**
Feng et al.[i]	2021	China	Targeted	63	--	52% (33)	20% (13)	-	-	-	8% (5)	-	12% (8)	-
Montal R et al.[ii]	2020	USA<br>Spain<br>Singapore	Whole genome expression<br>Targeted (150(	-	189 (144 x pCCA)	34.7% (52)	36.7% (55)	-	-	-	3% (5)	5.3% (8)	5% (8)	<1%
Weinberg et al.[iii]	2019	USA	Targeted	-	51	55% (28)	37% (18)	-	-	-	<5%	-	-	-
Chae et al.[iv]	2019	South Korea	Targeted	-	44	41% (18)	27% (12)	-	-	-	7% (3)	2% (1)	5% (2)	2% (1)
Lowery MA et al.[v]	2018	USA	Targeted	-	37	49% (18)	38% (14)	-	-	-	-	-	-	-
Wardell et al.[vi]	2018	Japan<br>Italy (46 targeted)	Whole exome<br>Whole genome<br>Targeted	109 (31)***	-	26%**	10%**	3%**	-	-	6%**	5%**	6%**	-
Jusakul et al.[vii]	2017	Singapore*<br>Thailand<br>Japan<br>South Korea,<br>USA,<br>Brazil	Whole genome whole exome Targeted	128*** (43)	-	35% (43)	9.8% (12)	-	-	2.4% (3)	4.1% (5)	5.7% (7)	3.3% (4)	0
Javle et al.[viii]	2016	USA	Targeted	-	57	40% (23)	42% (24)	-	-	-	-	7% (4)	-	0
Nakaumura et al.[ix]	2015	Japan *	Whole exome<br>Whole transcriptome	43	-	12% (5)	9% (4)	-	-	2%**	0	6%**	4%**	0
Churi et al.[x]	2014	USA	Targeted	-	20	45% (9)	40% (8)	-	-	-	5% (1)	10% (2)	5% (1)	5% (1)
Chan-On et al.[xi]	2013	Singapore*	Whole exome Targeted	-	33	9% (4)	11.1% (5)	-	-	-	-	-	-	-
Voss et al.[xii]	2013	USA	Targeted	-	27	-	22% (6)	-	-	-	-	-	-	-
Borger et al.[xiii]	2012	USA	Targeted	-	22	14% (3)	23% (5)	-	-	-	-	-	-	-

References

[i] Feng F, Wu X, Shi X, Gao Q, Wu Y, Yu Y et al. Comprehensive analysis of genomic alterations of Chinese hilar cholangiocarcinoma patients. International Journal of Clinical Oncology 2021 Jan 2. doi: 10.1007/s10147-020-01846-z. Online ahead of print.

[ii] Montal R, Sia D, Montironi C, Leow WQ, Esteban-Fabró R, Pinyol R et al. Molecular classification and therapeutic targets in extra-hepatic cholangiocarcinoma. Journal of Hepatology 2020;73(2):315-327.

[iii] Weinberg BA, Xiu J, Lindberg MR, Shields AF, Hwang JJ, Poorman K et al. Molecular profiling of biliary cancers reveals distinct molecular alterations and potential therapeutic targets. Journal of Gastrointestinal Oncology. 2019;10(4):652-662.

[iv] Chae H, Kim D, Yoo C, Kim KP, Jeong JH, Chang HM et al. Therapeutic relevance of targeted sequencing in management of patients with advanced biliary tract cancer: DNA damage repair gene mutations as a predictive biomarker. Europena Journal of Cancer 2019;120:31-39.

[v] Lowery MA, Ptashkin R, Jordan E, Berger MF, Zehir A, Capanu M et al. Comprehensive Molecular Profiling of Intrahepatic and Extrahepatic Cholangiocarcinomas: Potential Targets for Intervention. Clinical Cancer Research 2018;24(17):4154-4161.

[vi] Wardell CP, Fujita M, Yamada T, Simbolo M, Fassan M, Karlic R et al. Genomic characterization of biliary tract cancer identified driver genes and predisposing mutations. J Hepatology 2018; 68(5):959-969.

[vii] Jusakul A, Cutcutache I, Yong CH, Lim JQ, Huang MN, Padmanabhan N et al. Whole genome and epigenomic landscapes of etiologically distinct subtypes of cholangiocarcinoma Cancer Discovery 2017; 7(10):1116-1135.

[viii] Javle M, Bekaii-Saab T, Jain A, Wang Y, Kelley RK, Wang K et al. Biliary cancer: Utility of next-generation sequencing for clinical management. Cancer. 2016;122(24):3838-3847.

[ix] Nakamura H, Arai Y, Totoki Y, Shirota T, Elzawahry A, Kato M et al. Genomic sprectra of biliary tract cancer. Nature Genetics 2015;47(9):1003-10.

[x] Churi CR, Shroff R, Wang Y, Rashid A, Kang HC, Weatherly J et al. Mutation profiling in cholangiocarcinoma: prognostic and therapeutic implications. PLoS One. 2014;9(12):e115383.

[xi] Chan-On W, Nairismägi ML, Ong CK, Lim WK, Dima S, Pairojkul C et al. Exome sequencing identifies distinct mutational patterns in liver fluke-related and non-infection-related bile duct cancers. Nature Genetics 2013;45(12):1474-8.

[xii] Voss JS, Holtegaard LM, Kerr SE, Fritcher EG, Roberts LR, Gores GJ et al. Molecular profiling of cholangiocarcinoma shows potential for targeted therapy treatment decisions. Human Pathology 2013;44(7):1216-22.

[xiii] Borger DR, Tanabe KK, Fan KC, Lopez HU, Fantin VR, Straley KS et al. Frequent mutation of isocitrate dehydrogenase (IDH)1 and IDH2 in cholangiocarcinoma identified through broad-based tumor genotyping. Oncologist 2012;17(1):72-9.
