# Supplementary material for: Genomic profiling of idiopathic peri-hilar cholangiocarcinoma reveals new targets and mutational pathways
Source: Sci Rep. 2023 Apr 24;13:6681. doi: 10.1038/s41598-023-33096-0 (PMC10126102; doi:10.1038/s41598-023-33096-0)
Supplement: Supplementary file 2 — Supplementary Table 2. [file 41598_2023_33096_MOESM2_ESM.pdf]

Supplementary Table 2 – Germline mutations of conflicting or uncertain significance

Germline mutations identified in normal bile ducts with a variant allele frequency ≥30% and ≤70%, together with their respective amino acid changes and National Library of Medicine ClinVar significance are summarized in table 2. The non-biliary cancers in which these specific variants have been identified are also provided.

Gene	Mutation	Impact	Number of cases	ClinVar Significance	Cancer
APC	c.3386T>C	p.Leu1129Ser	1	Conflicting	Colorectal
ATM	c.146C>G	p.Ser49Cys	1	Conflicting	Breast
ATM	c.1229T>C	p.Val410Ala	1	Conflicting	Colorectal, Hereditary Cancer pre-disposing syndrome
BRCA1	c.4900A>G	p.Arg1634Gly p.Arg530Gly p.Arg1655Gly p.Arg1587Gly	3	Uncertain	Breast-ovarian
BRCA2	c.8567A>C	p.Glu2856Ala	1	Conflicting	Breast-ovarian
MEN1	c.512G>A	p.Arg171Gln	1	Conflicting	Hereditary cancer predisposing syndrome
MUTYH	c.930G>C	p.Gln310His	5	Uncertain	Colorectal
POLD1	c.2624G>A	p.Arg875His	1	Uncertain	Colorectal
RET	c.2071G>A	p.Gly691Ser	1	Conflicting	Hereditary cancer pre-disposing syndrome
TSC1	c.3103G>A	p.Gly1035Ser	1	Conflicting	Hereditary cancer pre-disposing syndrome
