# Supplementary material for: Genomic profiling of idiopathic peri-hilar cholangiocarcinoma reveals new targets and mutational pathways
Source: Sci Rep. 2023 Apr 24;13:6681. doi: 10.1038/s41598-023-33096-0 (PMC10126102; doi:10.1038/s41598-023-33096-0)
Supplement: Supplementary file 3 — Supplementary Table 3. [file 41598_2023_33096_MOESM3_ESM.pdf]

Supplementary Table 3 – Genes contained within significant SNV enriched pathways

Supplementary table 3 summarizes genes containing SNVs within each pathway of significance (<5% FDR) derived from gene set enrichment analysis. Pathways are arranged in descending order of significance.

**Pathway**	**FDR**	**FWER**	**Genes**
Dectin-2 family	0.001	0.008	MUC4<br>MUC5AC<br>MUC3A<br>CLEC4A<br>MUC5B<br>MUC13<br>PLCG2<br>MUC16<br>MUC17<br>CLEC6A<br>MUC6<br>MUC20<br>MUC12<br>MUC21<br>FCER1G<br>CLEC4E<br>MUC1<br>LYN<br>MUCL1<br>MUC15
Termination of O-Glycan Biosynthesis	0.003	0.019	ST6GALNAC4<br>MUC4<br>MUC5AC<br>MUC3A<br>MUC5B<br>MUC13<br>MUC16<br>ST3GAL3<br>MUC17<br>MUC6<br>MUC20<br>MUC12<br>ST6GALNAC2<br>ST3GAL4<br>MUC21<br>MUC1<br>ST6GALNAC3<br>MUCL1<br>MUC15

PD-1 signalling	0.007	0.09	CD4<br>PTPN6<br>HLA-DRB1<br>HLA-DQA2<br>CD3D<br>HLA-DQA1<br>HLA-DRA<br>TRBV12-3<br>TRAV29DV5<br>HLA-DRB5<br>CD274<br>CD3E<br>HLA-DPB1<br>TRBV7-9<br>HLA-DQB2<br>TRAV8-4<br>CD247
Translocation of ZAP-70	0.0095	0.126	CD4<br>HLA-DRB1<br>PTPN22<br>HLA-DQA2<br>CD3D<br>HLA-DQA1<br>HLA-DRA<br>TRBV12-3<br>TRAV29DV5<br>HLA-DRB5<br>CD3E<br>HLA-DPB1<br>TRBV7-9<br>HLA-DQB2<br>TRAV8-4<br>CD247<br>ZAP70
Phosphorylation of CD3 and TCR zeta chains	0.0098	0.149	CD4<br>HLA-DRB1<br>PTPN22<br>HLA-DQA2<br>CD3D<br>HLA-DQA1<br>HLA-DRA<br>PTPRJ<br>TRBV12-3<br>TRAV29DV5<br>HLA-DRB5<br>CD3E<br>HLA-DPB1

			TRBV7-9<br>HLA-DQB2<br>TRAV8-4<br>CD247
MET activates PTK2	0.01	0.177	PTK2<br>SRC<br>LAMA3<br>COL11A1<br>COL24A1<br>LAMA5<br>COL5A2<br>LAMA2<br>LAMA1<br>LAMC3<br>COL2A1<br>ITGA3<br>LAMB3<br>COL1A1<br>FN1<br>COL11A2<br>ITGA2<br>HGF<br>COL27A1<br>LAMB2<br>ITGB1<br>LAMA4<br>COL5A1<br>COL1A2<br>LAMC1<br>COL5A3<br>LAMB1
Collagen degradation	0.0152	0.279	PHYKPL<br>COL4A2<br>COL6A6<br>COL17A1<br>COL11A1<br>MMP1<br>COL16A1<br>COL4A6<br>MMP8<br>MMP9<br>COL5A2<br>COL6A1<br>COL13A1<br>COL2A1<br>COL6A5<br>MMP11

			COL4A3<br>MMP2<br>COL1A1<br>COL7A1<br>COL25A1<br>CTSK<br>COL9A3<br>COL11A2<br>COL23A1<br>COL26A1<br>ADAM9<br>COL19A1<br>COL6A2<br>COL4A1<br>COL12A1<br>COL4A4<br>COL14A1<br>CTSL<br>COL5A1<br>COL1A2<br>COL9A1<br>CTSB<br>COL6A3<br>COL18A1<br>MMP13<br>COL5A3<br>MMP14<br>CTSD
Collagen biosynthesis	0.0174	0.35	COL4A2<br>COL6A6<br>TLL1<br>COL20A1<br>PPIB<br>COL17A1<br>ADAMTS14<br>COL11A1<br>COL24A1<br>P3H1<br>COL22A1<br>COL16A1<br>COL4A6<br>COL5A2<br>COL6A1<br>COL13A1<br>COL2A1<br>COL6A5<br>SERPINH1

	0.019	0.416	COL4A3<br>COL1A1<br>ADAMTS3<br>COL7A1<br>COL25A1<br>CRTAP<br>PCOLCE2<br>COL28A1<br>P3H2<br>COL9A3<br>COL11A2<br>COL23A1<br>COL26A1<br>COL19A1<br>COL6A2<br>COL27A1<br>COL4A1<br>COL12A1<br>COL4A4<br>COL14A1<br>COL5A1<br>COL1A2<br>COL9A1<br>COL21A1<br>COL6A3<br>COL18A1
NCAM1 interactions	0.019	0.416	COL4A2<br>COL6A6<br>CACNA1S<br>CACNA1C<br>COL5A2<br>COL6A1<br>CACNA1G<br>COL2A1<br>AGRN<br>COL6A5<br>COL4A3<br>CACNA1H<br>COL9A3<br>CNTN2<br>CACNB4<br>COL6A2<br>CACNB2<br>COL4A1<br>GFRA1<br>COL4A4<br>CACNA1D

			COL5A1<br>COL9A1<br>ARTN<br>COL6A3<br>NRTN<br>COL5A3<br>CACNB3
Class I MHC folding and assembly	0.021	0.508	CANX<br>SEC24A<br>PDIA3<br>SEC23A<br>SEC31A<br>HLA-A<br>HLA-G<br>HLA-B<br>ERAP1<br>HLA-E<br>HLA-F<br>SEC24D<br>SEC24B<br>HLA-C<br>TAPBP<br>TAP2<br>SEC24C<br>SEC13<br>CALR<br>TAP1<br>SAR1B<br>ERAP2
TH1TH2 pathway	0.022	0.464	NR1I2<br>IL2RA<br>CD86<br>HLA-DRB1<br>HLA-DRA<br>IFNGR2<br>HLA-DRB5<br>IL12RB2<br>IL4R
NOTCH HLH Transcription	0.0297	0.742	NCOR2<br>HDAC4<br>NOTCH3<br>MAML3<br>HDAC5<br>MAML2<br>HDAC9<br>CREBBP<br>HDAC7

			NOTCH1<br>NOTCH4<br>RBPJ<br>HDAC10<br>TBL1X<br>MAML1<br>NCOR1<br>KAT2B<br>HDAC3<br>NOTCH2
NRAGE signals death through JNK	0.039	0.868	MCF2L<br>BAD<br>ARHGEF16<br>OBSCN<br>NET1<br>MCF2<br>ECT2<br>ARHGEF1<br>VAV2<br>PREX1<br>ARHGEF2<br>TIAM2<br>ARHGEF10<br>ARHGEF10L<br>ABR<br>ARHGEF26<br>TRIO<br>ARHGEF7<br>VAV1<br>SOS2<br>ARHGEF40<br>ARHGEF3<br>KALRN<br>NGEF<br>ARHGEF37<br>ITSN1<br>ARHGEF4<br>PLEKHG5<br>ARHGEF12<br>AKAP13<br>FGD4<br>ARHGEF33<br>BCL2L11<br>ARHGEF19<br>SOS1<br>ARHGEF5
Receptor	0.045	0.901	PTPRF

type tyrosine protein phosphatase			PPFIBP1<br>PTPRS<br>PTPRD<br>PPFIBP2<br>SLITRK2<br>PPFIA3<br>IL1RAP<br>PPFIA1<br>SLITRK6<br>IL1RAPL1<br>NTRK3<br>IL1RAPL2<br>SLITRK4<br>LRRC4B<br>PPFIA4
Intestinal immune network	0.046	0.899	ITGA4<br>AICDA<br>TNFRSF17<br>CXCL12<br>CD86<br>HLA-DRB1<br>TNFRSF13B<br>HLA-DQA2<br>TGFB1<br>HLA-DQB1<br>AC005840.1<br>HLA-DQA1<br>CXCR4<br>CCL27<br>IL10<br>HLA-DRA<br>MAP3K14<br>TNFSF13<br>HLA-DRB5<br>CCR10<br>HLA-DOA<br>MADCAM1<br>IL15<br>HLA-DPB1<br>IL5<br>CD80<br>CCL28<br>IL15RA<br>ICOS
Interferon gamma signalling	0.046	0.934	SOCS1<br>VCAM1<br>CIITA

			JAK2<br>PTPN6<br>IRF7<br>PRKCD<br>GBP6<br>CAMK2A<br>TRIM5<br>HLA-DRB1<br>HLA-A<br>TRIM34<br>HLA-G<br>HLA-DQA2<br>HLA-B<br>HLA-DQB1<br>CAMK2B<br>HLA-E<br>HLA-DQA1<br>TRIM10<br>TRIM62<br>HLA-H<br>TRIM68<br>HLA-DRA<br>TRIM6<br>IFNGR2<br>CD44<br>GBP1<br>HLA-F<br>TRIM22<br>TRIM31<br>HLA-DRB5<br>GBP2<br>TRIM35<br>OAS2<br>JAK1<br>SP100<br>SUMO1<br>TRIM48<br>AC004551.1<br>OAS3<br>GBP3<br>TRIM29<br>HLA-C<br>PIAS1<br>TRIM14<br>PTPN2<br>PTPN1<br>GBP5

			CAMK2G<br>HLA-DPB1
ARF6 trafficking	0.047	0.921	ITGA4<br>CTNND1<br>INS<br>KLC1<br>IL2RA<br>TSHR<br>ITGA1<br>ITGAV<br>AVPR2<br>ITGA11<br>ITGA6<br>ITGA3<br>ITGA5<br>EXOC4<br>SLC2A4<br>EXOC3<br>CLTC<br>ITGA10<br>EXOC6<br>SCAMP2<br>CTNNA1<br>ITGA2<br>VAMP3<br>EDNRB<br>MAPK8IP3<br>DNM2<br>ITGB1<br>EXOC7<br>EXOC2<br>ACAP1<br>PLD2<br>ITGA7<br>PIP5K1C<br>CDH1<br>ITGA9<br>SPAG9<br>ITGA8<br>PLD1
SEMA4D induced migration	0.047	0.92	MYH9<br>SEMA4D<br>MYL12B<br>MYH14<br>LIMK2<br>ERBB2<br>MYH10

			ARHGEF12<br>RHOC<br>RHOA<br>ROCK2<br>LIMK1<br>MYH11<br>PLXNB1
PITX2	0.048	0.942	DVL1<br>KAT5<br>EP300<br>APC<br>TRRAP<br>LEF1<br>MED1<br>CREBBP
RHO GTPases activate ROC	0.049	0.953	PPP1R12B<br>MYH9<br>MYL12B<br>PPP1R12A<br>CFL1<br>MYH14<br>LIMK2<br>MYH10<br>PPP1CB<br>RHOC<br>RHOA<br>ROCK2<br>LIMK1<br>MYH11
Mismatch repair	0.049	0.953	MSH2<br>RPA1<br>MSH3<br>POLD3<br>POLD1<br>PMS2<br>EXO1<br>MSH6<br>POLD2
