# Supplementary material for: Genomic profiling of idiopathic peri-hilar cholangiocarcinoma reveals new targets and mutational pathways
Source: Sci Rep. 2023 Apr 24;13:6681. doi: 10.1038/s41598-023-33096-0 (PMC10126102; doi:10.1038/s41598-023-33096-0)

Supplementary Figure 1: Principle component analysis (cluster analysis) of exon containing SNVs in idiopathic peri-hilar cholangiocarcinoma tumors. Principle component analysis using a sparse approach was undertaken to ascertain how closely patients clustered together on the basis of mutations within exon containing regions in tumor tissue. Following removal of two outliers on initial PCA, all remaining patients clustered closely together on the basis of exons containing SNVs. Each sequential PCA followed this pattern whereby two further outliers were generated following removal of the previous two outliers.

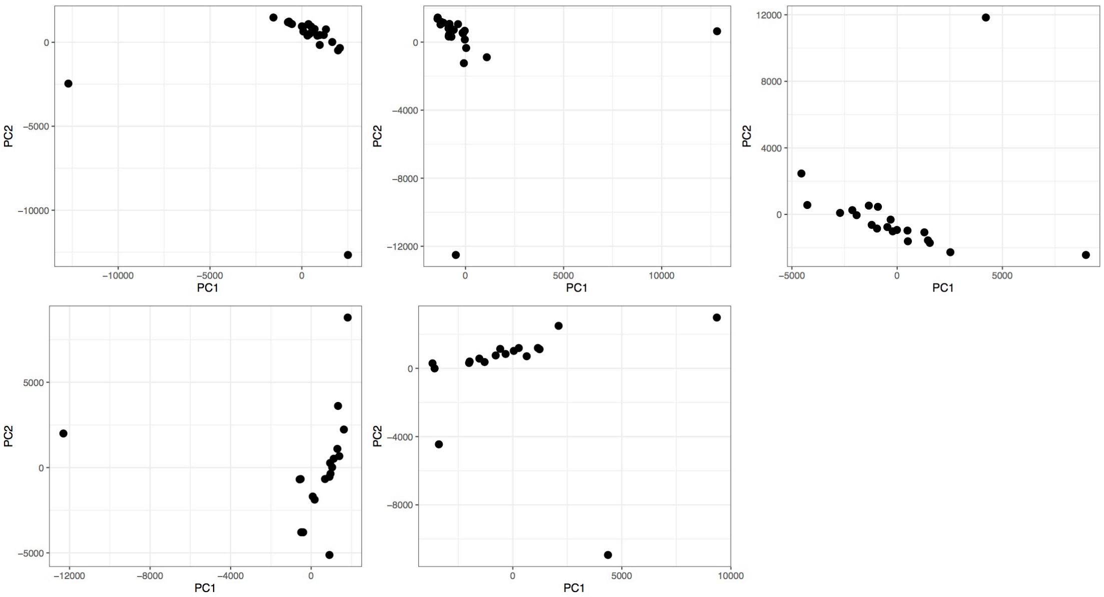

Supplement: Supplementary file 4 — Supplementary Figure 1. [file 41598_2023_33096_MOESM4_ESM.pdf]
