# Supplementary figures and images for: Genomic profiling of idiopathic peri-hilar cholangiocarcinoma reveals new targets and mutational pathways
Source: Sci Rep. 2023 Apr 24;13:6681. doi: 10.1038/s41598-023-33096-0 (PMC10126102; doi:10.1038/s41598-023-33096-0)

TP53 Frameshift

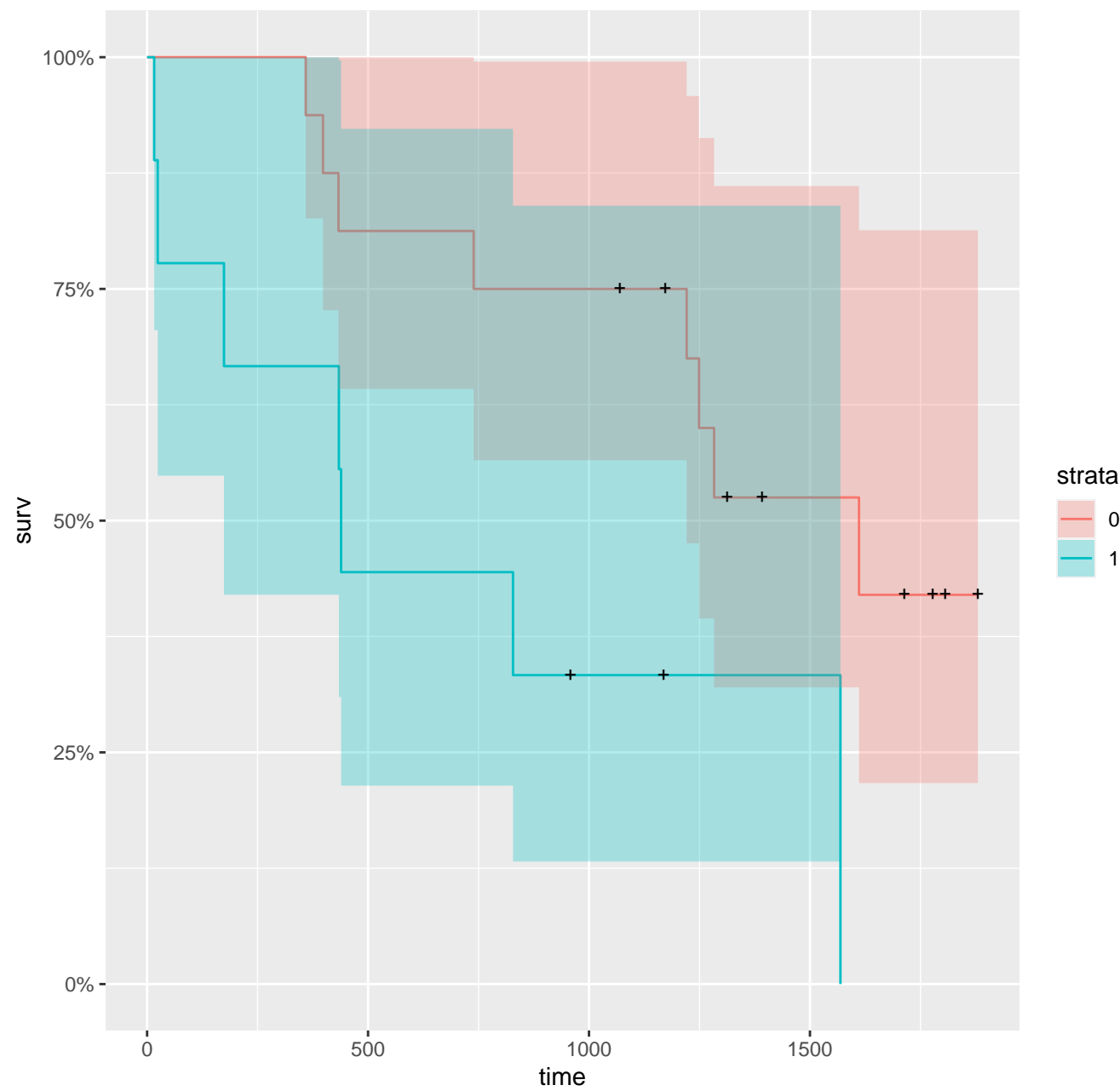

Supplement: Supplementary file 5 — Supplementary Figure 2. [file 41598_2023_33096_MOESM5_ESM.pdf]
