# Supplementary material for: Genomic profiling of idiopathic peri-hilar cholangiocarcinoma reveals new targets and mutational pathways
Source: Sci Rep. 2023 Apr 24;13:6681. doi: 10.1038/s41598-023-33096-0 (PMC10126102; doi:10.1038/s41598-023-33096-0)
Supplement: Supplementary file 6 — Supplementary Legends. [file 41598_2023_33096_MOESM6_ESM.docx]

**Supplemental Figure 2**

Kaplan-Meier suvival curve for estimating the probability of overall survival (days) in those patients with or without a TP53 frameshift mutation in the whole exome sequencing cohort.  The presence of a TP53 frameshift mutation in tumor conferred a worse overall survival (HR 3.33, p<0.033).
